# Supplementary material for: Bovine Pancreatic RNase A: An Insight into the Mechanism of Antitumor Activity In Vitro and In Vivo
Source: Pharmaceutics. 2022 May 30;14(6):1173. doi: 10.3390/pharmaceutics14061173 (PMC9229056; doi:10.3390/pharmaceutics14061173)
Supplement: Supplementary file 1 [file pharmaceutics-14-01173-s001.zip › pharmaceutics-1734156-supplementary.pdf]

Table S1. Sequence of the primers used for reverse transcription (RT) of miRNAs.

Name	Sequences of the RT primer, 5′ → 3′
RT mir-31	GTCGTATCCAGTGCAGGGTCCGAGGTATTCGCACTGGATACGACCAGCTATGCC
RT mir-21	GTCGTATCCAGTGCAGGGTCCGAGGTATTCGCACTGGATACGACTCAACATCAG
RT mir-145a	GTCGTATCCAGTGCAGGGTCCGAGGTATTCGCACTGGATACGACAGGGATTCCT
RT mir-155	GTCGTATCCAGTGCAGGGTCCGAGGTATTCGCACTGGATACGACTGGATACGAC
RT mir-10b	GTCGTATCCAGTGCAGGGTCCGAGGTATTCGCACTGGATACGACCACAAATTCG
RT let7-g	GTCGTATCCAGTGCAGGGTCCGAGGTATTCGCACTGGATACGACAACTGTACAA
RT U6	GTCGTATCCAGTGCAGGGTCCGAGGTATTCGCACTGGATACGACAAAAATATGGA ACG

Table S2. Sequences of the forward and reverse primers for qPCR.

Name	Sequences of the Specific Primer, 5′ → 3′
mir-31-F	AGGCAAGATGCTGGCA
mir-21-F	AGACTAGCTTATCAGACTGA
mir-145a-F	AGGTCCAGTTTTCCCAGGA
mir-155-F	ACTTAATGCTAATTGTGATAGG
mir-10b-F	TACCCTGTAGAACCGAA
let7-g-F	AACGCTGAGGTAGTAGTTTGT
U6-F	CTCGCTTCGGCAGCACA
Universal-R	GTGCAGGGTCCGAGGT
E-cadherin (Cdh1) -F	GAAGAAGGAGGTGGAGAAGAAG
E-cadherin (Cdh1) -R	CATCAGGATTGGCAGGACG
Vimentin -F	CTCCTACGATTCACAGCCAC
Vimentin -R	GAGCCACCGAACATCCTG
Fibronectin -F	CTTTGTGGTCTCATGGGTCTC
Fibronectin -R	AGCAGGTCAGGAATGTTCAC
Tjp-1 -F	CGAGTTGCAATGGTTAACGG
Tjp-1 -R	CAGGATCTGGGTGACTTACAG
HPRT -F	CTGGTGAAAAGGACCTCTCGAAG
HPRT -R	CCAGTTTCACTAATGACACAAACG

Table S3. The penetration efficiency of the RNase A-biotin conjugate in B16 and HeLa cells.

Concentration of the conjugate, μM	B16		Hela	
	Fluorescent cells, %	Fluorescence intensity, RFU	Fluorescent cells, %	Fluorescence intensity, RFU
0 (control)	0.62	2.9	0.47	16.8
1	3.3	5.0	1.7	20.3
5	13.0	5.0	3.5	21.3
10	41.5	8.1	9.7	30.4
20	68.2	13.2	17.2	30.9

Table S4. The sequences of studied miRNAs in mouse and human.

	**mouse, 3′→5′**	**human, 3′→5′**
miR-21a	uagcuuaucagacugauguuga	uagcuuaucagacugauguuga
mir-145a	guccaguuuucccaggaaucccu	guccaguuuucccaggaaucccu
mir-31	aggcaagaugcuggcauagcug	aggcaagaugcuggcauagcu
mir-10b	uacccuguagaaccgaauuugug	uacccuguagaaccgaauuugug
let-7g	ugagguaguaguuuguacaguu	ugagguaguaguuuguacaguu
miR-155	uuaaugcuaauugugauaggggu	uuaaugcuaaucgugauagggguu

100% homology of mmu-miR and hsa-miR. Data were taken from MirBase (www.mirbase.org).

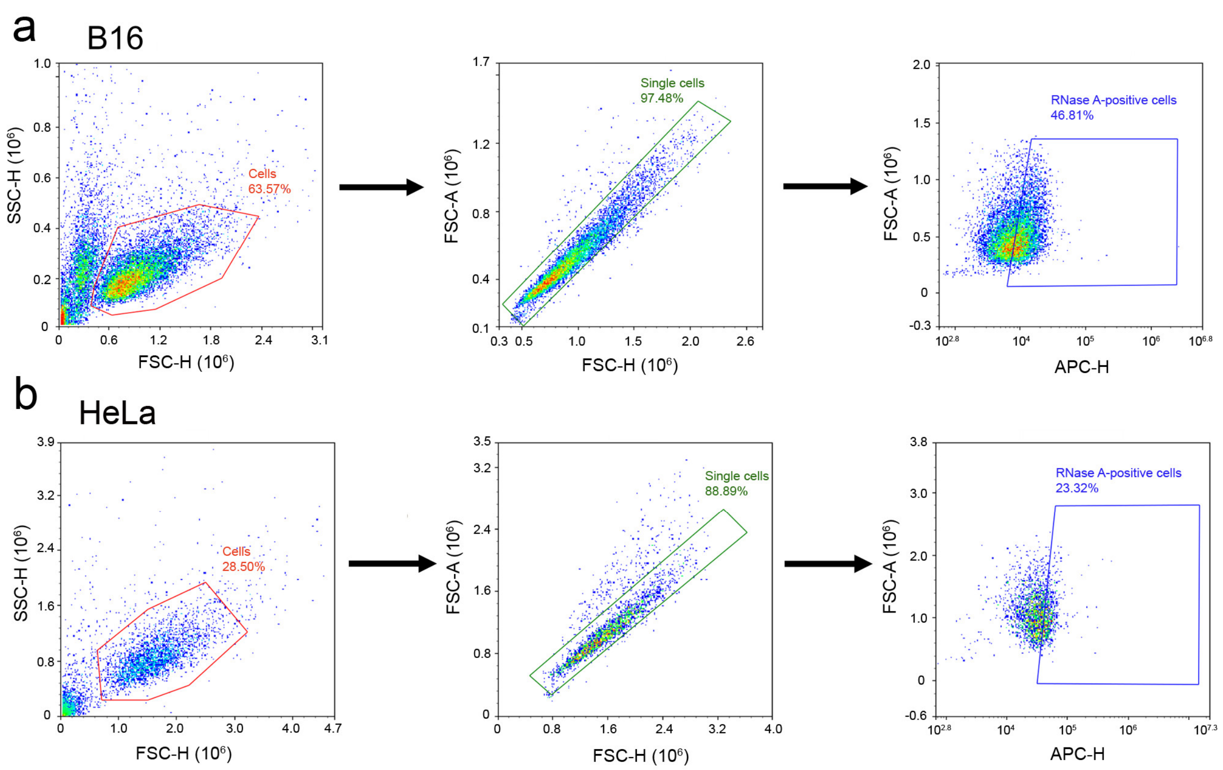

Figure S1. Gating strategy. (a) B16 cells. (b) HeLa cells. B16 and HeLa cells were incubated in the presence of RNase A-biotin conjugate at concentrations 10 and 20 μM, respectively, for 4 h. Data of flowcytometry.

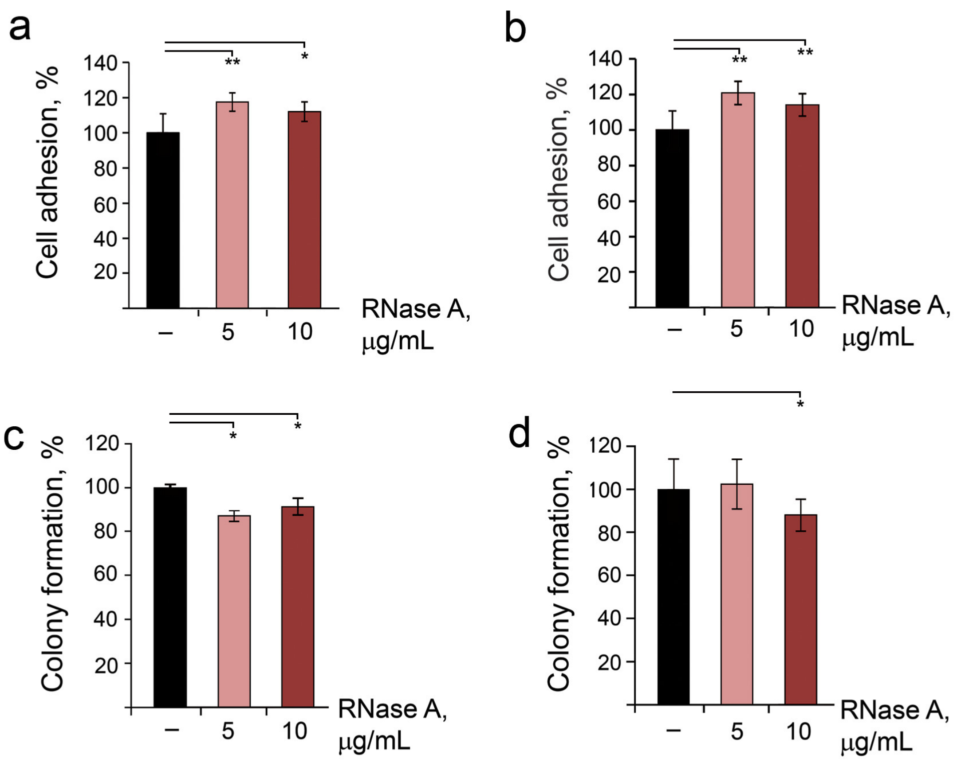

Figure S2. The effect of RNase A on adhesion and colony formation of B16 and HeLa cells. (a,b) Adhesion of B16 and HeLa cells to plates, respectively, after cell treatment with RNase A (5 and 10 µg/mL). The extent of cell adhesion was quantified by MTT assay. (c,d) Clonogenic activity of B16 and HeLa cells after treatment with RNase A, respectively. B16 and HeLa cells were treated by RNase A (5 and 10 µg/mL) for 8 and 14 days, respectively. The assessment of the colony formation was carried out using a ColonyArea ImageJ plugin. Data are presented as mean ± S.E.M. Data were statistically processed using Student´s T-test; *p<0.05, **p<0.01, control (non-treated cells). All experimental points were run in triplicate for statistical analysis.

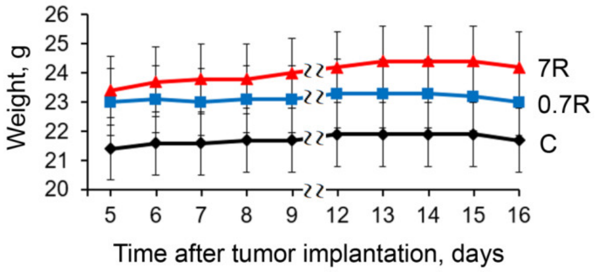

Figure S3. Weight of mice with B16 during the experiment. C - mice with B16 treated with saline buffer; 0.7R and 7R – mice with B16 treated with RNase A at the doses of 0.7 and 7 μg/kg, respectively.
